# Skin microbiota variation among Indian monozygotic twins

**DOI:** 10.7717/peerj.21208

**Published:** 2026-05-28

**Authors:** Renuka Potbhare, Manjari Jonnalagadda, Leo Lahti, Richa Ashma

**Affiliations:** 1 Department of Zoology, Savitribai Phule Pune University, Pune, Maharashtra, India; 2 Symbiosis School for Liberal Arts, Symbiosis International (Deemed University), Pune, Maharashtra, India; 3 Department of Computing, University of Turku, Turku, Finland

**Keywords:** Skin microbiota, Diversity, 16S sequencing, Genetic relatedness, Geography

## Abstract

**Background:**

The human skin microbiota is shaped by host-specific factors such as age, diet, geography and environment. However, the interplay between these, especially host genetics and skin microbiota composition remains largely unexplored in Indians. Monozygotic twins share 100% of their genetic makeup, thereby providing a unique opportunity to investigate the associations between genetic relatedness, environmental variation, and microbiota composition.

**Methods:**

We collected axillary sweat samples from thirteen monozygotic twin pairs (*n* = 26) and sequenced V3–V4 regions of the16S rRNA gene on Illumina.

**Result:**

In the present study *Firmicutes* (Bacillota in the newly adopted LPSN/NCBI nomenclature), Proteobacteria (Pseudomonadota), and *Actinobacteria* (Actinomycetota) were observed as the most prevalent phyla. The relative abundances of most prevalent phyla and genera varied within monozygotic twin pairs. We found that geographical location was associated with skin microbiota diversity, with alpha diversity showing a borderline association, whereas beta diversity (Jaccard index) differed significantly across the three locations. However, neither diet nor gender was significantly associated with alpha or beta diversity. Additionally, pairwise comparisons based on the Jaccard index revealed significant differences at the genus level between twins *vs* siblings, and siblings *vs* unrelated individuals; however, these differences were not significant using the Bray–Curtis dissimilarity index.

**Conclusion:**

This pilot study expands the current knowledge of the skin microbiota and its association with genetic relatedness and other potential confounders. We demonstrated the association of twins’ skin microbiota composition and diversity with geographical location; however, further studies with a larger representation of monozygotic (MZ), dizygotic (DZ) and sibling-parent pairs living in different households are needed to understand the interplay between genetics, environmental variation, and skin microbiota diversity, and composition.

## Introduction

Skin harbours diverse microorganisms, providing a dynamic environment for viruses, bacteria, and fungi, collectively known as the microbiota. These skin bacterial communities maintain homeostasis and protect the body from pathogens (Byrd, Belkaid & Segre, 2018). The resident (core) bacteria are permanent skin inhabitants, while transient bacteria are temporary visitors and often unable to establish long-term colonisation. Core bacterial communities can be present in all individuals, though their abundance varies depending on skin physiology, such as moisture level, sebum production and pH (Grice et al., 2009; Kong, 2011). It is known that the composition and distribution of skin microbiota differ from person to person (Fierer et al., 2010). Furthermore, the alteration of microbiota is also driven by intrinsic and extrinsic host factors (Ruuskanen et al., 2022) such as age (Kim et al., 2022; Ratanapokasatit et al., 2022), gender (Robert et al., 2022), diet (Wallen-Russell, Gijsberts-Veens & Wallen-Russell, 2021; Mahmud et al., 2022), geography (Potbhare et al., 2022; Ruuskanen et al., 2021), and lifestyle (Moitinho-Silva et al., 2021; Kortekaas et al., 2024). Additionally, living in a particular geographical area shapes the individual microbiota composition through climate, light exposure, and moisture content (Potbhare et al., 2022).

Despite the extensive research on intrinsic and extrinsic factors that govern skin microbiota composition, the role of genetics remains unexplored. While our previous studies on Indian joint families focused on the associations between confounding factors such as age, diet, and geographical location with skin microbiota, the effect of genetic relatedness on skin microbiota remains unexplored (Potbhare et al., 2025; Chaudhari et al., 2020).

Gut microbiota studies on genetically related individuals have demonstrated that host genetics influence microbial communities (Wang et al., 2016) and genetic factors shape microbiota composition (Benson et al., 2010; Goodrich et al., 2014; Goodrich et al., 2016). A study by Goodrich et al. (2016) found that certain gut taxa are heritable. Furthermore, skin microbiota heritability studies have observed the passing of bacterial taxa across generations (Si et al., 2015; Potbhare et al., 2022). Although, the functional properties of gut microbiota differ from those of the skin microbiota, the characteristic healthy skin microbiota communities are essential for understanding confounder associations and their functionality (Joos et al., 2024). Analysing monozygotic twins’ microbiota provides a unique opportunity to better elucidate the interplay among genetics, environment, and variation in skin microbiota.

In the present study, we analysed 13 pairs of monozygotic twins and characterised their bacterial communities using 16S rRNA gene sequencing targeting the V3–V4 region. Given the close genetic kinship shared by monozygotic twins, we hypothesized that they would share more similar skin microbiota profiles than unrelated individuals. To further explore the association of genetic relatedness, we also compared skin microbiota composition between twins, siblings (from different families) and genetically unrelated individuals. Likewise, we analyse the skin microbiota diversity and its association with genetic relatedness alongside other potentially confounding factors such as cohabitation, geographical location, gender, and diet.

## Materials & methods

### Ethical approval, study consent, and history forms

The present study follows the ethical guidelines issued by the ICMR, Government of India and was approved by the Institutional Human Ethics Committee (IEC) of Savitribai Phule Pune University, Pune (Letter No. SPPU/IEC/2019/57). The ICMR guidelines are aligned with the Declaration of Helsinki and the research has been conducted in accordance with it. All cohabiting twins were informed about the purpose of the study, the sample collection procedure, and written consent was obtained from each participant, in accordance with the Declaration of Helsinki. We collected individual history forms, including a questionnaire on medication history (metabolic disorders like diabetes mellitus, cardio-vascular disease, thyroid hormone dysregulation, kidney malfunction *etc.*), lifestyle variables (diet, age, gender, geographical location of the participants), alcohol and smoking consumption habits, antibiotic treatment (long term medication related to any skin disorders), and cosmetic (soap, body lotion, deodorants) usage, during the sampling process. The participants were enrolled only if they reported no medication history of metabolic disorders, no long-term use of antibiotics (at least six months), no alcohol or smoking habits, and no dermatological diseases. The twins who met the stringent criteria were considered healthy and recruited in the present study. Furthermore, participants were also instructed to avoid axilla shaving, bathing, consumption of food items such as onion, garlic, (and therefore obligatory non-vegetarians were not considered for the study), and the use of soap, deodorants, and moisturisers for 24 hrs prior to sample collection.

### Participant enrollment and sample collection

**Figure 1:**
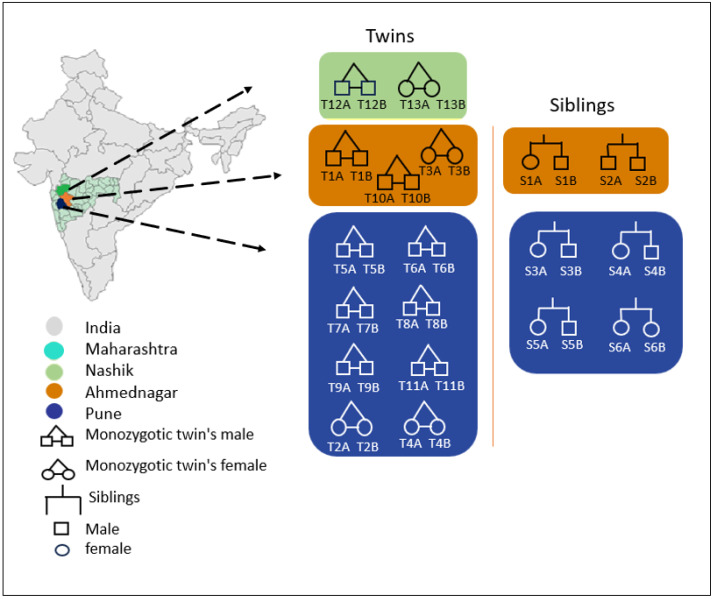
Graphical summary of the recruited twins and siblings along with their geographical locations. The color represents the geographical location; shapes are denoted in the left panel of the figure. The labelling of the twins and siblings based on sample metadata.

A total of 13 healthy, monozygotic twin pairs (*n* = 26) ranging in age from 16 to 28 years were recruited for this study. The selected male (*n* = 18) and female (*n* = 8) volunteers were residents of three cities of Maharashtra, India, viz., Pune (altitude: 1840 ft, longitude: 73°51′19.26″E; latitude: 18°31′10.45″N), Ahmednagar (altitude: 2129 ft, longitude: 74°44′58.53″E; latitude: 19°5′42.75″N) and Nashik (altitude: 1916 ft; longitude: 73°47′27.46″E; longitude; 19°59′50.17″N). The recruited twins had similar dietary habits, were vegetarian or mixed to mitigate the impact of diet on skin microbiota. Additionally, to minimise the effect of the environment, the cohabiting twin pairs were selected. Although efforts have been made to match individuals based on general living conditions, specific environmental factors like hygiene or cleanliness practices could have contributed to the microbiota variation, as before sampling, participants were asked to avoid axilla shaving, bathing, and use of soap, deodorants, and moisturisers. The anthropological data of participants is given in Table S1 and a graphical summary of the participants is shown in Fig. 1. We used state boundary data from the GADM database (version 4.1, http://www.gadm.org) for the map outlines.

The samples were collected in three studied cities-Nashik, Ahmednagar, and Pune on the same dates and times. In each city, the sampling was conducted at a single place after screening twins. Twins who fulfilled the inclusion criteria were contacted within the next 2 days at a designated place in their respective cities. All the sampling was performed during fixed morning hours (10 am to 12 pm) before bathing the participants. This schedule helped minimise variation in sampling method, sampling day and time, city-specific climatic conditions such as temperature, sweating, and clothing material, while collecting the samples. This approach enhanced the rigour of the study by controlling for environmental variations.

Sweat samples from both axillae were collected using sterilised cotton swabs moistened with autoclaved phosphate-buffered saline (PBS) (1X). Participants were asked to rolled the moistened cotton swabs multiple times in the axillary region after physical activity to ensure sweat was collected on the swab. The sweat samples were collected in triplicate from each participant on the same day of sampling. Due to low skin bacterial biomass, DNA was pooled while sequencing.

### Bacterial DNA extraction and 16S rRNA gene sequencing

Bacterial DNA extraction, purification, quantitation, and gel electrophoresis were performed as described in our previous study (Potbhare et al., 2022). In brief, bacterial cells were first lysed with a lysis buffer containing of 0.5 M EDTA, 0.5 M Tris–HCl, 0.1% Triton X, and 8% sucrose. The lysate was then treated with Lysozyme, RNAse and Proteinase K, ensuring cell wall rupture followed by RNA and protein digestion. The DNA was then purified using the standard phenol extraction method described by Sambrook, Fritsch & Maniatis (1989). The resulting nucleic acids were precipitated by repeated washes of 70% and 100% ethanol. Further, the presence of DNA was confirmed by agarose gel electrophoresis and on a nanodrop spectrophotometer at 260 nm and 280 nm absorbance.

Extracted DNA was amplified by Polymerase Chain Reaction (PCR) using 2x KAPA HiFi HotStart ReadyMix^®^ (KAPA Biosystems, Boston, USA). The universal bacterial primers targeting V3–V4 regions of 16S rRNA gene (Forward Primer: 5′ TCGTCGGCAGCGTCAGATGTGTATAAGAGACAGCCTACGGGNGGCWGCAG-3′ Reverse Primer: 5′ GTCTCGTGGGCTCGGAGATGTGTATAAGAGACAGGACTACHVGGGTATCTAATCC-3′) were used (Klindworth et al., 2013). The total reaction volume of 25 uL was used for each PCR amplification PCR reaction using the following thermal conditions: initial denaturation at 95 °C for 3 min followed by 25 cycles of 95 °C for the 30s; 55 °C for the 30s; and 72 °C for 30s. The final extension was set at 72 °C for 5 min. We have used distilled water as a negative control for DNA extraction and the PCR thermal cycle. The amplified products were then purified with AMPure XP beads using PureLinkTM PCR purification kit^®^ (Invitrogen, Waltham, MA, USA). This was followed by index PCR of 8 cycles and adapter attachment using the Nextera XTIndex Kit^®^ (Illumina, San Diego, CA, USA) under the same conditions as the amplification procedure. The amplicons were then purified using PureLink™ PCR purification kit^®^ (Invitrogen, Waltham, MA, USA) and subjected to quality assessment using the Qubit 2.0 fluorometer (Life Technologies, Waltham, MA, USA). The final purified products were pooled and V3–V4 regions of the 16S rRNA gene were sequenced on the Illumina Mi-seq platform using 2*250 bp paired chemistry.

### Bioinformatics analysis

The FASTQ files containing raw sequences were obtained from the 16S Illumina BaseSpace hub. The adapter sequences were removed using the FASTQ toolkit (version 2.2.5) and reads were trimmed to an average quality score of 30, maintaining a minimum read length of 180 bp and a maximum of 200 bp to remove low-quality reads. Generated High-quality (HQ) reads were denoised and merged using DADA2 (version 1.36.0) to remove chimeric sequences. A total of 858 amplicon sequence variant (ASV) were obtained from HQ reads after removal of singletons. Taxonomic classification to ASVs was performed by assigning RefSeq, the RDP reference database (16S v3 May 2018 DADA2) obtained from the 16S Metagenomic application (version 1.1.3) of Illumina BaseSpace (Callahan et al., 2016). We also noted that the nomenclature of microbial taxa has recently been updated (Lloyd & Tahon, 2022); our present work uses the old nomenclature because the reference database version used at the time the taxonomic profiling for this dataset was conducted.

Further, we used distilled water as the negative control rather than autoclaved PBS. Although this approach identifies reagent and laboratory-associated contaminants, it limits the ability to detect potential contaminants arising from the sample collection process. Future studies incorporating collection controls would provide a more robust evaluation of collection-derived contamination. We identified 10ASVs exclusively in the negative control (Table S2) representing approximately 1.18% of the total 858 ASVs.

A total of 2,093,416 reads passed a quality score of 30, ranging from a minimum of 46,007 to a maximum of 119,944 reads per sample. Altogether, 847 ASVs were obtained from quality-passed sequences with no singletons present. The pre-processed data was then imported into R/Bioconductor TreeSummarizedExperiment format (Huang et al., 2021).

### Statistical analysis

The skin bacterial community diversity and composition were analysed using *mia* (version 1.6.0) and visualised with *miaViz* (version1.6.0) (Borman et al., 2025). The overall taxonomic composition of the most abundant taxa based on the geographical location of twins was carried out by setting up a detection threshold 0.1%, ensuring inclusion of taxa above this level and avoiding rare taxa. Additionally, a 20% of prevalence threshold was applied, ensuring each taxon must be present in at least 20% of all samples. These filtering criteria were applied only for visualization of community composition and not used for alpha and beta diversity (Bray–Curtis and Jaccard index) analysis, ensuring that diversity metrics were calculated using a full dataset.

Further, alpha diversity association with confounders including diet, gender, and geographical location were estimated using the Shannon diversity index. We used rarefaction (niter = 100) to ensure robustness of the result (Schloss, 2024). The *p*-values were adjusted using FDR (False Discovery Rate). FDR < 0.05 was considered significant, and <0.1 was considered borderline significant. Microbiota community dissimilarity was estimated using the Bray–Curtis and Jaccard indices, with 100 rarefaction iterations. These two indices were chosen because they provide complementary aspects of community variation, as Jaccard is based on presence/absence, whereas Bray–Curtis additionally considers the abundance levels. We used Bray–Curtis as the primary index, and Jaccard index to further confirm that the results are robust to index choice. Principal Coordinates Analysis (PCoA) was performed to visualise the similarity of skin microbiota between samples. Differences in community composition (*i.e.* beta diversity) was compared between groups using PERMANOVA with 999 permutations using the mia R package. To minimise the effect of genetic relatedness among twin pairs, we analyzed each set of twin pairs separately, keeping only one randomly selected twin per pair to assess the association between confounders and microbiota composition. Each set consisted of 13 non-repeated members, with only one member from each twin pair. The alpha and beta diversity analyses were carried out at the genus level. Additionally, we also carried out differential abundance analysis using ANCOM-BC2 (Lin & Das, 2024) to determine taxa that were significantly different among twins based on geographical location, however no taxa reached statistical significance after correction for multiple testing and covariate effects (no data reported).

We explored dissimilarities in skin microbiota community composition by comparing twins with non-twins. The pairs were matched by geographical location to control for potential confounding factors. Additionally, we investigated skin microbiota dissimilarities between twins, siblings, and unrelated individuals. The sibling data was sourced from our recently published manuscript (Potbhare et al., 2025). These recruited siblings followed exactly the same methodology as the current twin’s study, including, skin sampling type (moist), sampling site (both axilla), sampling procedure (sterile cotton swab), DNA extraction method (phenol extraction) and same Illumina Miseq platform for sequencing of V3–V4 region of *16S rRNA* gene using 250*2 bp pair-end chemistry. Since, both the twins and siblings samples were run on a single flow cell, batch variations were minimised. The same Illumina BaseSpace platform was used for data preprocessing and bioinformatic analysis. Likewise, only age-matched, and cohabiting siblings were recruited for a comparative study with twins, so as to limit the age and cohabitation effect. Further, the unrelated non-cohabiting pairs were made by selecting one member from each twin and sibling pair. All the analysis were carried out at the genus level to investigate major shifts in the skin microbiota. The samples included in both studies were collected simultaneously and analysed using the same method described above.

## Results

### Analysis of skin microbiota composition of twins across three geographical locations

**Figure 2:**
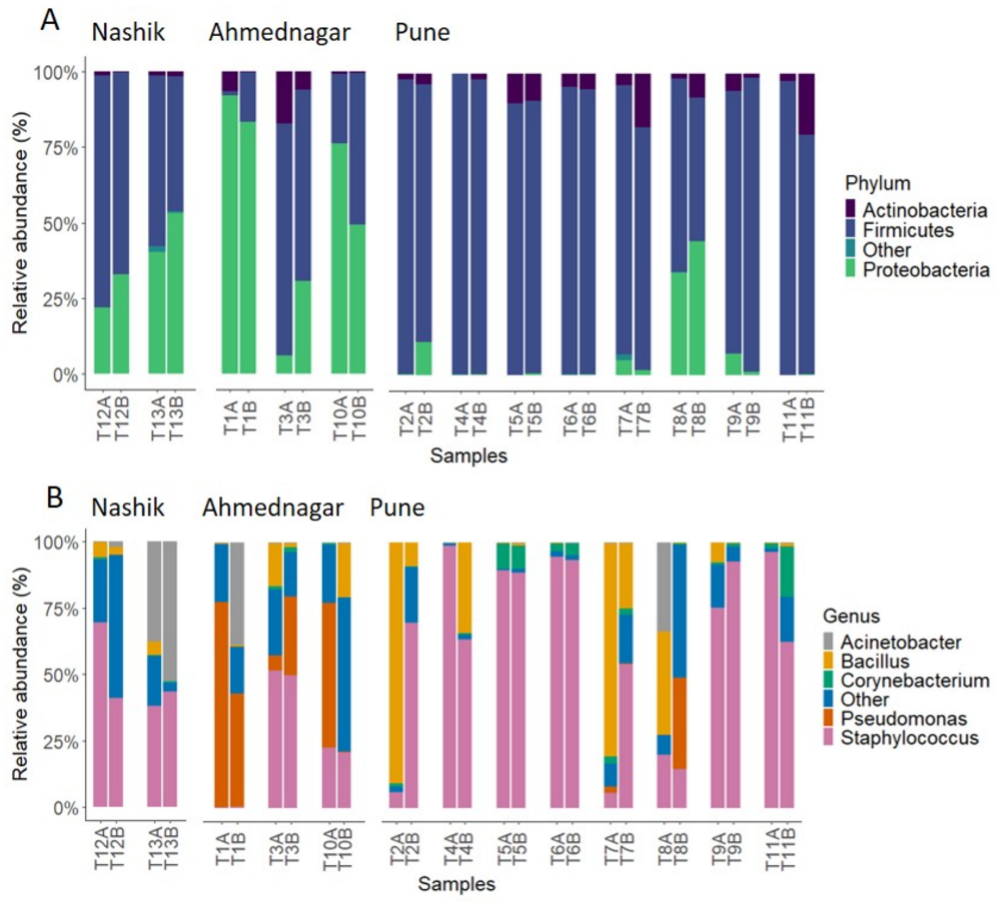
Relative abundances of most prevalent (A) phyla and (B) genera at 0.1% detection threshold and 20% prevalence. *X*-axis arranged by sample ID.

The analysis of skin microbiota composition in our study showed the presence of 36 bacterial phyla, 285 families, and 848 genera in total across the three sampling locations. Among these, twin pairs were most similar at the phylum level, as evident in Fig. 2A; however, the genus-level variation was higher among the twins (Fig. 2B).

The prevalence and relative abundance of the top three phyla were presented in Table 1. In summary, *Firmicutes (Bacillota* in the newly adopted LPSN/NCBI nomenclature*)* was detected as the most prevalent phylum across all three geographical locations with the highest mean relative abundance in Pune (87%), followed by Nashik (61%) and Ahmednagar (38.4%). Contrastingly, *Proteobacteria (Pseudomonadota*) showed high prevalence and mean relative abundance (100%, 56.2%) in Ahmednagar and Nashik (100%, 37.1%) while considerably lower levels in Pune (100%, 6.7%). On the other hand, *Actinobacteria (Actinomycetota)* were detected across three geographical locations with 100% prevalence but represented only a small portion of the community with the highest mean relative abundance in Pune (7%) and Ahmednagar (5.2%) compared with Nashik (1.2%). The rest of the phyla with prevalence <20% and a 0.1% detection threshold were grouped together as “other”. They were frequently detected in Nashik and Pune (0.5%) and least in Ahmednagar (0.01%).

Likewise, the prevalence and relative abundances of the top six genera were described in Table 2. In brief, *Staphylococcus* was the most prevalent (100%) genus across all locations, with the highest mean relative abundance in Pune (64.1%), and Nashik (47.9%), followed by Ahmednagar (24.3%). *Bacillus* was detected as the second most prevalent (100–81.2%) genus in Pune (mean relative abundance 18%), then Ahmednagar (6.5%) and Nashik (3.5%). *Acinetobacter* showed moderate prevalence and mean relative abundance in Nashik (75%, 22.9%) and Ahmednagar (50%, 6.5%), but was detected less in Pune (12.5%, 2.1%). Further, *Corynebacterium* was reported frequently, particularly in Pune (93.7%), with mean relative abundance generally low across three locations. *Pseudomonas* was less prevalent and less abundant in Nashik (0.01%, 0.04%) but relatively common in Ahmednagar (83.3%, 35%) and Pune (31.2%, 2.3%). The “other” genera were rare and grouped together based on a <20% prevalence and 0.1% detection threshold. They were detected in almost all samples across each location, with a mean relative abundance ranging from 25–27% in Nashik and Ahmednagar, and only 10% in Pune.

**Table 1:** The most prevalent phyla at 0.1% detection threshold and 20% prevalence reported in the study.

	**Nashik**		**Ahmednagar**		**Pune**	
Phylum	Prevalence (%)	Mean relative abundance (%)	Prevalence (%)	Mean relative abundance (%)	Prevalence (%)	Mean relative abundance (%)
*Actinobacteria*	100	1.2	100	5.2	100	7.0
*Firmicutes*	100	61.0	100	38.4	100	87.0
*Proteobacteria*	100	37.1	100	56.2	93.7	6.7
*Other*	20	0.5	20	0.01	0.5	0.5

**Table 2:** The most prevalent genera at 0.1% detection threshold and 20% prevalence reported in the study.

	**Nashik**		**Ahmednagar**		**Pune**	
Genus	Prevalence (%)	Mean relative abundance (%)	Prevalence (%)	Mean relative abundance (%)	Prevalence (%)	Mean relative abundance (%)
*Acinetobacter*	75.0	22.9	50.0	6.5	12.5	2.1
*Bacillus*	100	3.5	83.3	6.5	81.2	18.0
*Corynebacterium*	75.0	0.5	66.6	0.6	93.7	3.5
*Pseudomonas*	0.01	0.04	83.3	35.0	31.2	2.3
*Staphylococcus*	100	47.9	100	24.3	100	64.1
*Other*	100	25.1	100	27.1	100	10

### Diversity analysis

#### Alpha diversity analysis

**Figure 3:**
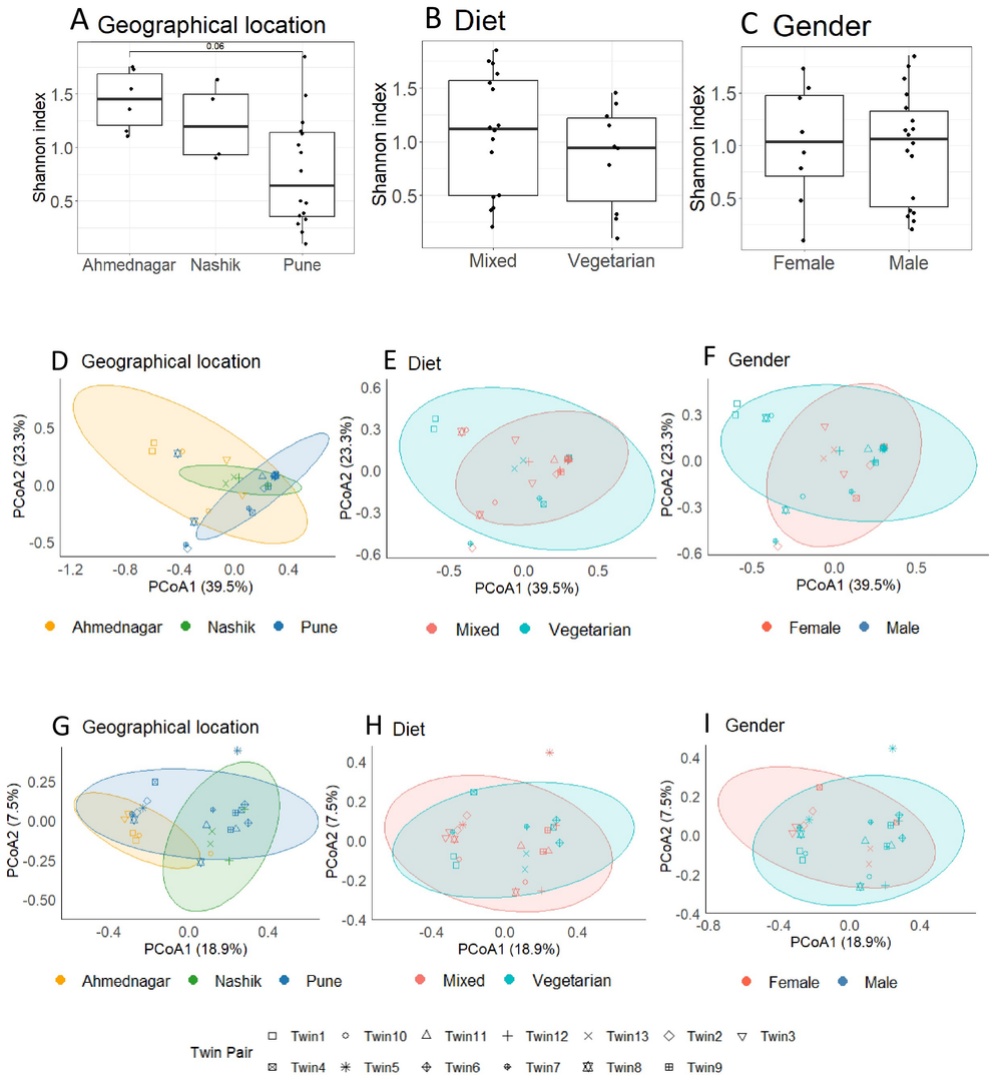
Diversity analysis. Alpha diversity analysis for confounding factors using Shannon index (A) Geographical location (Kruskal–Wallis; FDR = 0.06); (B) Diet (FDR = 0.40); (C) Gender (FDR = 0.95). Principal Coordinates Analysis using Bray–Curtis dissimilarity at the genus level, where samples are colored according to the distribution of potential confounders and shaped by twin pairs (D) Geographical location (PERMANOVA *p* = 0.06); (E) Diet (*p* = 0.7); (F) Gender (*p* = 0.29). Principal Coordinates Analysis using Jaccard dissimilarity (G) Geographical location (*p* = 0.01**); (H) Diet (*p* = 0.58); (I) Gender (*p* = 0.20). The p-value indicates level of significance<0.001***, <0.01**, <0.05*.

We evaluated the skin microbiota association with confounding factors by comparing alpha diversity between groups using the Kruskal–Wallis test (Fig. 3A). The geographical location had a borderline association with alpha diversity (FDR = 0.06). Diet (FDR = 0.40), or gender (FDR = 0.95) did not reveal a significant skin microbiota association.

#### Beta diversity analysis

The principal coordinate analysis (PCoA) based on the Bray–Curtis index provided a visual illustration of the association between community composition and—geographical location, diet, and gender (Fig. 3B). The samples with similar microbial profiles clustered closely together. The first two axes of PCoA accounted for a substantial proportion of variance—62.8% (PC1 = 39.5%, PC2 = 23.3%). We observed borderline clustering of microbiota profiles between twin pairs based on geographical location (Bray–Curtis; *p* = 0.06), but no clear clusters were observed by diet (*p* = 0.7) and gender (*p* = 0.29), which PERMANOVA further confirmed (Table 3A).

We further analyzed our beta diversity results using the Jaccard index to assess the sensitivity of the results to the selected dissimilarity index. The PCoA suggested partial clustering of twins based on geographical location only and no clear separation by diet and gender were observed (Fig. 3C). The total variance explained by PC1 was 18.9% and PC2 explaining 7.5%, summing to 26.4%. PERMANOVA confirmed a significant association of geographical location (Jaccard, *p* = 0.01**) and a non-significant association of diet (*p* = 0.58) and gender (*p* = 0.20) (Table 3B).

Collectively, our alpha and beta diversity analysis (using both Bray–Curtis and Jaccard indices) highlighted a borderline to significant association between skin microbiota composition and the twins’ geographical location. This finding underscores the impact of environmental factors on skin microbiota diversity, and it is a crucial step towards understanding the complex interplay between genetic makeup and the environment in shaping the skin microbiota.

**Table 3:** PERMANOVA for confounding factors Geographical location; Diet; and Gender with (A) Bray Curtis index and (B) Jaccard Index.

**Confounding factor**	R^2^	*p*-value
(A)		
Geographical location	2.1	0.06
Diet	0.64	0.70
Gender	1.22	0.29

(B)		
Geographical location	1.51	0.01^**^
Diet	0.90	0.58
Gender	1.51	0.20

**Notes.**

The *p*-value indicates level of significance <0.001***, <0.01**, <0.05*.

### Skin microbiota analysis of twin pairs

**Figure 4:**
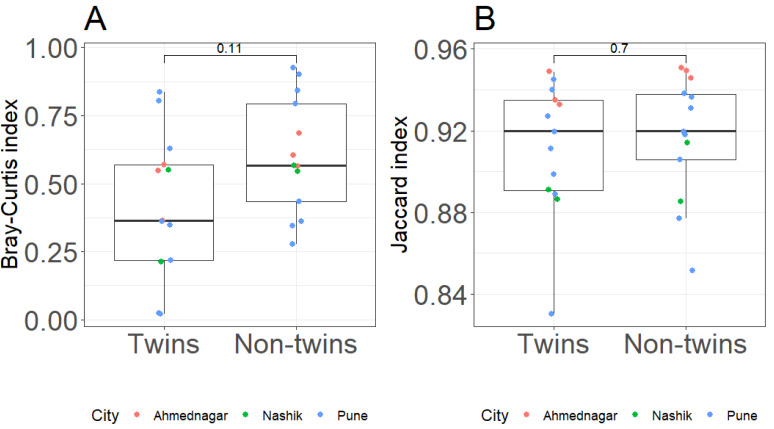
Comparative analysis of twins and non-twin at genus level with (A) Bray–Curtis index (Kruskal–Wallis test, *p* = 0.11) (B) Jaccard index (Kruskal–Wallis test, *p* = 0.7). Note: The *Y*-axis for Jaccard index is shown on truncated (non-zero) scale.

To understand the association of genetic relations we compared twins with non-twins in terms of skin microbiota dissimilarity. Non-twin pairs were made by randomly selecting one member from each twin pair and comparing it with a randomly chosen, unrelated twin pair. We did not observe a significant difference in skin microbiota between twins (genetically identical) *vs.* non-twins (genetically non-identical) using Bray–Curtis (Kruskal–Wallis test, *p* = 0.11) or Jaccard index (Kruskal–Wallis test, *p* = 0.7) (Figs. 4A–4B).

### Skin microbiota analysis of twins, siblings and unrelated individuals

**Figure 5:**
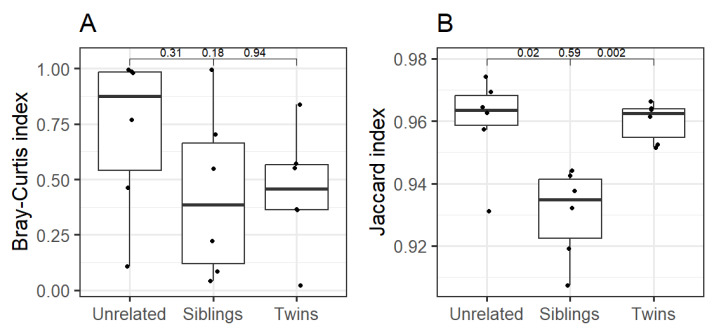
Comparative analysis between unrelated individuals, siblings, and twins at genus level with (A) Bray–Curtis index (Kruskal–Wallis test) (B) Jaccard index (Kruskal–Wallis test). Note: The *Y*-axis for Jaccard index is shown on truncated (non-zero) scale.

We further compared twins, siblings and unrelated individuals to investigate the genetic associations underlying differences in skin microbiota. This analysis included six pairs of twins, siblings (from genetically unrelated families) and unrelated individuals taken from our recently published dataset (Potbhare et al., 2025). To ensure balanced comparisons across groups, the number of unrelated pairs was matched with the available siblings and twin pairs. The unrelated pairs were generated by randomly selecting one member of a twin pair and age-matched siblings from different families. Altogether, six pairs per group (twins, siblings, and unrelated individuals) were included in the intra-pair similarity analysis. The comparison based on Bray–Curtis index revealed that twins and sibling pairs had, on average, a similar level of dissimilarity (Kruskal–Wallis test, *p* = 0.94). This also held between twins and unrelated individuals (*p* = 0.18), and siblings and unrelated individuals (*p* = 0.31) (Fig. 5A). On the other hand, the same comparison using the Jaccard index highlighted significant skin microbiota difference between twins *vs* siblings (Kruskal–Wallis test, *p* = 0.002) and siblings *vs* unrelated individuals (*p* = 0.02), while twins *vs* unrelated individuals did not show a significant difference (*p* = 0.59) (Fig. 5B).

Altogether, twins and siblings had, on average, more similar microbiota composition than unrelated individuals. This could be due to differences in cohabitation patterns, life histories, or in the similarity of their genetic make-up.

## Discussion

Our study reports the variation in skin microbiota in genetically identical twin pairs of Indian origin across three geographical locations viz. Pune, Ahmednagar, and Nashik cities of Maharashtra. We observe a distinctive microbiota composition between twin pairs, typically dominated by phyla *Firmicutes (Bacillota), Proteobacteria (Pseudomonadota), and Actinobacteria (Actinomycetota)* with variation in their abundances across geographical locations. Our results are consistent with the previous studies on the facial microbiota of twins from the USA, wherein *Firmicutes (Bacillota)* were highly abundant, followed by *Proteobacteria (Pseudomonadota),* and *Actinobacteria (Actinomycetota)* (Zaidi et al., 2018). These three dominant phyla were also reported on the skin of Korean twins (Si et al., 2015). We observed *Staphylococcus, Corynebacterium, Bacillus,* and *Pseudomonas* as the most prevalent genera with alterations in the abundance in each of the studied twin pairs. These genera were also found to be abundant in earlier studies of the skin microbiota, with varying relative abundances across samples (Skowron et al., 2021; Oh et al., 2014; Cundell, 2018; Shen et al., 2023). Furthermore, we observed that the composition of genera classified as “other” differed in their relative abundances on the twins’ skin.

Despite genetic similarities, environmental factors are most related to skin microbiota composition (Si et al., 2015; Potbhare et al., 2025) which are concordant with the diversity analysis performed in the present study and show borderline differences in alpha diversity (within twin pairs), and borderline (Bray–Curtis index) to statistically significant (Jaccard index) differences in beta diversity (between different twin pairs). These results were varied across two commonly used dissimilarity indices in the present study. The observed differences in the geographical location could be due to variations in the climatic conditions of Pune, Ahmednagar, and Nashik; however, these districts are in close proximity, and differ marginally in the climatic parameters such as humidity, rainfall amounts, and urbanisation status (Potbhare et al., 2022). The study by Freire et al. (2020) on twins’ oral microbiotas suggested that the environment primarily shapes the microbiota compared to host genetics. Hence, understanding environmental exposure provides valuable insights into predicting risk for early diseases, which would aid in developing future diagnostic tools.

An individual’s genetic makeup is known to influence the diversity and composition of skin microbiota (Byrd, Belkaid & Segre, 2018; Dimitriu et al., 2019). However, earlier research on the association between the skin microbiota and genetics focused mainly on genetically unrelated individuals (Potbhare et al., 2022; Leung, Wilkins & Lee, 2015). Only a few studies have investigated the influence of genetics on family datasets (Potbhare et al., 2022) and twin datasets (Si et al., 2015). The comparative study by Si et al. (2015) on the skin microbiota between Monozygotic (MZ) and Dizygotic (DZ) twins revealed that MZ twins share a more similar microbiota than DZ twins, suggesting genetics might play a role in shaping the skin microbial communities, although this is difficult to distinguish from other confounding factors. Further, their findings on heritability and additive environmental effects on skin microbiota reported that *Corynebacteria* and *Brevibacterium* were heritable in twins (Si et al., 2015). Although we observed no significant difference in the twins *versus* non-twins comparison using Bray–Curtis, it was evident that twins share, on average, a microbiota similar to that of non-twins. This could be attributed to genetic relatedness or cohabitation, as these pairwise comparisons were made within the same geographical location, limiting the potential contribution of geographical location and helping to evaluate the association with genetic relatedness and cohabitation. That said, our current study design does not allow for distinguishing genetic relatedness from confounding factors such as co-habitation and is a limitation that should be addressed in future research, where in it would be useful to conduct skin microbiota studies on twins by controlling environmental conditions, along with other intrinsic (age, gender) and extrinsic (diet, lifestyle) factors and larger sample size to further elucidate the association with genetic relatedness. In contrast, when comparison was carried out using the Jaccard index, no difference was observed between twins and non-twins skin microbiota; this could be due to the operation of these indices where Bray–Curtis is a quantitative measure (abundance) and Jaccard is a non-parametric measure based on presence/absence. This may explain the differences in the results; while the groups may share similar taxa, variation in the abundance level can affect comparisons using the Bray–Curtis index.

Our comparative analysis of twins, siblings and unrelated groups based on the presence/absence-based Jaccard index showed significant differences between unrelated *vs.* siblings and twins *vs.* siblings, while the abundance-based Bray–Curtis showed non-significant differences. Taken together, the results suggest that genetically related groups (twins and siblings) shared more similar taxa than unrelated groups, while the abundance levels can vary considerably. Further, although both indices showed lower diversity among genetically related groups, differences in the abundance and types of microbiota could be attributed to lifestyle factors like indoor and outdoor activity. In the present study, monozygotic twins (MZ) were compared with siblings from the same family rather than dizygotic twins (DZ) for logistical and cost-efficiency reasons. While siblings share ∼50% of their genetic makeup, like DZ twins on average, they do not share a prenatal environment, age, or early life exposure that is intrinsic to twin pairs. Thus, the classical twin studies rely on comparing MZ and DZ twin pairs who share similar early life exposure but differ in genetic relatedness; therefore, they are better suited to disentangle the genetics and environmental contributions.

Twin microbiota research has demonstrated the heritability of susceptible bacteria in dermatological conditions such as acne and psoriasis. Aldrich et al. (2015) investigated the genetic underpinnings of common skin diseases by studying identical and fraternal twins. Their findings suggested that both heredity and environment contribute equally to the development of rosacea. Furthermore, their extended research reported a significant correlation between facial microbiota and rosacea in twins compared with non-twins, suggesting that genetic composition plays a vital role in shaping microbiota (Aldrich et al., 2015). Another study of Caucasian female monozygotic and dizygotic twins reported that acne is a highly heritable skin condition with significant additive genetic effects (Bataille et al., 2002).

Moreover, numerous twin studies have concentrated on gut microbiota and dysbiosis, exploring how the host’s dietary patterns influence the heritable aspects of the microbiota (Goodrich et al., 2016). However, the role of host genetics in the skin microbiota has yet to be studied.

This is the first study investigating axillary skin microbiota variation in Indian monozygotic twins. A more extensive analysis with a larger number of monozygotic (MZ) and dizygotic (DZ) twins, and a study design specifically targeted to disentangle genetic effects, will be necessary to understand the influence of genetics on the skin microbiota. Furthermore, analysing twins provides an opportunity to understand how genetic relatedness is linked to this variation, which could support recommendations on personalised medicine, targeted treatments for skin diseases and forensic investigations. Twin’s microbiota studies also serve as a reference for future research on genetically related datasets, as their genetic and environmental similarities provide a unique platform for investigating other aspects, such as diet, age, and epigenetics.

## Limitations

The present study informs that genetics and environment both contribute to skin microbiota composition; however, we emphasize that our findings are indicative, considering the small sample size of twin pairs. We could recruit twins who meet the stringent sampling criteria of cohabitation, healthy, non-smoking, non-alcoholic, age-matched, with similar dietary habits, *etc.* Further, in this study, we could control for personal and household hygiene practices for the selected twin pairs, which may have potentially contributed to skin microbiota variation between twin pairs. Nonetheless, data have been analysed using standard, robust statistical methods used in this field, providing valuable leads for future research.

Although our study design used the standard sequencing depth as other skin microbiota studies, we mainly limited our analysis to the genus and higher taxonomic levels due to the resolution of the measurement platform (Johnson et al., 2019; Hiergeist et al., 2023). Sequencing a full-length 16S rRNA gene could help capture finer taxonomic detail, including low-prevalence, and low-abundance ASVs. In addition, subsequent work should explore the functional aspects of microbial community variation, using methods such as PICRUSt (Douglas, Beiko & Langille, 2018) or PICRUSt2 (Douglas et al., 2020) or MicFunPred (Mongad et al., 2021).

##  Supplemental Information

10.7717/peerj.21208/supp-1 Supplemental Information 1 Demographics of recruited twins and siblings

10.7717/peerj.21208/supp-2 Supplemental Information 2 ASVs detected in the negative control

## Additional Information and Declarations

### Data Availability

The following information was supplied regarding data availability: The code and data is available at Github and Zenodo: - https://github.com/Renuka-3/Twins_microbiome
- Renuka Potbhare. (2026). Renuka-3/Twins_microbiome: Twins microbiome updated analysis (1.1). Zenodo. https://doi.org/10.5281/zenodo.14590933
The sequences are available at the European Nucleotide Archive: - PRJEB44216: ERS6234991–ERS6235003
- PRJEB79728: ERS20952628–ERS20952640
- PRJEB62887: ERS15583999, ERS15584000, ERS15584010, ERS15584011, ERS15584017, ERS15584018, ERS15584027, ERS15584028, ERS15584030, ERS15584031.
