# Supplementary material for: Skin microbiota variation among Indian monozygotic twins
Source: PeerJ. 2026 May 28;14:e21208. doi: 10.7717/peerj.21208 (PMC13222544; doi:10.7717/peerj.21208)
Supplement: Supplemental Information 1 [file peerj-14-21208-s001.docx]

Supplementary table 1: Demographics of recruited twins and siblings.

| Pair ID | Zygosity | Gender | Diet | Location |
| --- | --- | --- | --- | --- |
| Twin1 | Monozygotic | Male | Vegetarian | Ahmednagar |
| Twin2 | Monozygotic | Female | Mixed | Pune |
| Twin3 | Monozygotic | Female | Mixed | Ahmednagar |
| Twin4 | Monozygotic | Female | Vegetarian | Pune |
| Twin5 | Monozygotic | Male | Mixed | Pune |
| Twin6 | Monozygotic | Male | Vegetarian | Pune |
| Twin7 | Monozygotic | Male | Vegetarian | Pune |
| Twin8 | Monozygotic | Male | Vegetarian | Pune |
| Twin9 | Monozygotic | Male | Mixed | Pune |
| Twin10 | Monozygotic | Male | Mixed | Ahmednagar |
| Twin11 | Monozygotic | Male | Mixed | Pune |
| Twin12 | Monozygotic | Male | Mixed | Nashik |
| Twin13 | Monozygotic | Female | Vegetarian | Nashik |
| Sibling1 | - | Female, Male | Vegetarian | Ahmednagar |
| Sibling2 | - | Female, Male | Mixed | Pune |
| Sibling3 | - | Male, Male | Mixed | Ahmednagar |
| Sibling4 | - | Female, Male | Vegetarian | Pune |
| Sibling5 | - | Female, Male | Mixed | Pune |
| Sibling6 | - | Female, Female | Vegetarian | Pune |
