# Supplementary material for: Skin microbiota variation among Indian monozygotic twins
Source: PeerJ. 2026 May 28;14:e21208. doi: 10.7717/peerj.21208 (PMC13222544; doi:10.7717/peerj.21208)
Supplement: Supplemental Information 2 [file peerj-14-21208-s002.docx]

| **ASVs** | **Number of reads** | **% of reads** |
| --- | --- | --- |
| Bacillus firmus (D16268) | 21 | 0.033 |
| Acinetobacter indicus (HM047743) | 14 | 0.022 |
| Acinetobacter variabilis (KP278590) | 11 | 0.017 |
| Pseudomonas stutzeri (AF094748) | 4 | 0.006 |
| Calothrix sp. (AM230678) | 1 | 0.002 |
| Thiolamprovum pedioforme (Y12297) | 1 | 0.002 |
| Bacillus licheniformis (CP000002) | 1 | 0.002 |
| Solimonas aquatica (EU303271) | 1 | 0.002 |
| Lysobacter ruishenii (GU086401) | 1 | 0.002 |
| Exiguobacterium sibiricum (CP001022) | 1 | 0.002 |

Supplementary table 2: The table of ASVs detected in the negative control.
